# Well-Being of US Military Veterans

**DOI:** 10.1001/jamanetworkopen.2023.46709

**Published:** 2023-12-07

**Authors:** Peter J. Na, Ian C. Fischer, Alex H. Krist, Harold S. Kudler, Dilip V. Jeste, Robert H. Pietrzak

**Affiliations:** 1 VA Connecticut Healthcare System, West Haven; 2 Department of Psychiatry, Yale School of Medicine, New Haven, Connecticut; 3 Department of Family Medicine and Population Health, Virginia Commonwealth University, Richmond; 4 Department of Psychiatry and Behavioral Sciences, Duke University, Durham, North Carolina; 5 Department of Veterans Affairs Mid-Atlantic Mental Illness Research, Education, and Clinical Center, Durham, North Carolina; 6 Global Research Network on Social Determinants of Mental Health and Exposomics, La Jolla, California; 7 National Center for PTSD, VA Connecticut Healthcare System, West Haven, Connecticut; 8 Department of Social and Behavioral Sciences, Yale School of Public Health, New Haven, Connecticut

## Abstract

This survey study analyzed data from a nationally representative sample of US veterans to examine ratings and correlates of well-being.

## Introduction

The National Academies of Science, Engineering, and Medicine recently published a report^1^ that suggests a transition from a reactive disease-oriented medical care system to one that prioritizes disease prevention, health, and well-being. The report evaluated and made recommendations for the US Department of Veterans Affairs’ national implementation of a Whole Health initiative, which emphasizes a person-centered, values-based approach to health and well-being.^2^ To help inform this initiative, this cross-sectional survey study analyzed data from a nationally representative sample of US veterans to examine ratings and correlates of well-being.

## Methods

A total of 2435 veterans participated in the 2022 National Health and Resilience in Veterans Study (NHRVS; eMethods in Supplement 1). This study followed the AAPOR reporting guidelines, and participants provided electronic informed consent. The ethics committee of VA Connecticut Healthcare System approved the NHRVS.

Well-being was assessed using the 12-item Flourishing Measure,^3^ which assesses 6 domains of well-being—happiness and life satisfaction, mental and physical health, meaning and purpose, character and virtue, close social relationships, and financial and material stability (Cronbach α = 0.91). Ratings range from 0 to 10 (higher scores indicate higher well-being) and are averaged to yield an overall measure of well-being (eTable in Supplement 1).

Analyses of variance were conducted between June 10 and 12, 2023, to compare well-being ratings by age, sex, and race and ethnicity. Pearson correlations (Bonferroni-corrected α = .05/33 = .002) and multivariable linear regression (2-sided *P* < .05) were then conducted to identify significant correlates of well-being. Interaction terms were incorporated into this analysis to evaluate the potential role of protective psychosocial characteristics (eg, purpose in life) in moderating associations between negative correlates of well-being (eg, physical health conditions). Analyses were conducted using SPSS, version 29 (IBM Corp).

## Results

The sample of 2435 veterans had a mean (SD) age of 63.3 (13.9) years and was predominantly male (n = 2177 [92.3%, weighted]) and White, non-Hispanic (n = 2027 [79.5%, weighted]) (Table). Younger, female, and Hispanic veterans had the lowest well-being scores, with the largest magnitude difference observed between veterans aged 65 years or older (mean age, 74.5 years) and veterans aged 21 to 54 years (mean age, 44.1 years) (effect size difference, Cohen *d* = 0.77) (Figure).

**Table.** Sample Characteristics and Correlates of Well-Being Among US Veterans^a^

Characteristic	Weighted mean (SD) or No. (weighted %)	Bivariate correlation analyses, *r*	Multivariable linear regression (*R*^2^ = 0.51), β
Sociodemographic characteristics			
Age, weighted mean (SD), y	63.3 (13.9)	0.29^b^	0.19^c^
Sex, No. (weighted %)			
Male	2177 (92.3)	0.08^b^	0.01
Female	258 (7.7)		
Race and ethnicity, No. (weighted %)			
White, non-Hispanic	2027 (79.5)	0.02	NA
Black, non-Hispanic	164 (10.2)		
Hispanic	164 (6.0)		
Other, non-Hispanic^d^	80 (4.3)		
College graduate or higher education, No. (weighted %)	1139 (35.2)	0.08^b^	−0.02
Married or partnered, No. (weighted %)	1768 (74.4)	0.16^b^	0.05^e^
Retired, No. (weighted %)	1368 (46.0)	0.13^b^	0.03
Household income ≥$60 000, No. (weighted %)	1501 (62.5)	0.12^b^	0.02
Military characteristics			
Enlisted or commissioned vs drafted, No. (weighted %)	2134 (88.1)	−0.08^b^	0
Combat veteran, No. (weighted %)	843 (35.9)	−0.02	NA
≥10 y in military, No. (weighted %)	913 (38.4)	0.02	NA
Rank or pay grade in military	E-5	0.10^b^	0.01
Positive effect of military on life, weighted mean (SD) score	2.1 (1.3)	0.25^b^	0.03^f^
Health characteristics			
Physical health difficulties, weighted mean (SD) score	0 (1.0)	−0.30^b^	−0.14^c^
Physical exercise, weighted mean (SD) score	1.2 (0.9)	0.08^b^	0.01
Adverse childhood experiences, weighted mean (SD) score	1.4 (1.9)	−0.26^b^	−0.03
Cumulative trauma burden, weighted mean (SD) score	8.8 (8.1)	−0.09^b^	0.01
Military sexual trauma, No. (weighted %)	162 (5.9)	−0.15^b^	−0.02
Lifetime posttraumatic stress disorder, No. (weighted %)	222 (9.8)	−0.28^b^	0.01
Lifetime major depressive disorder, No. (weighted %)	324 (13.5)	−0.32^b^	−0.03
Lifetime suicide attempt, No. (weighted %)	74 (3.2)	−0.17^b^	−0.01
Lifetime alcohol use disorder, No. (weighted %)	964 (40.2)	−0.19^b^	0
Lifetime drug use disorder, No. (weighted %)	263 (11.3)	−0.18^b^	−0.03^f^
Lifetime nicotine use disorder, No. (weighted %)	411 (15.8)	−0.12^b^	−0.01
Personality, weighted mean (SD) score			
Extraversion	3.8 (1.5)	0.31^b^	0.04^f^
Agreeableness	5.1 (1.2)	0.34^b^	0.02
Conscientiousness	5.9 (1.1)	0.34^b^	0.02
Emotional stability	5.4 (1.3)	0.47^b^	0.10^c^
Openness to experiences	4.8 (1.2)	0.24^b^	0.01
Psychosocial factors, mean (SD) score			
Protective psychosocial characteristics	0 (1.0)	0.64^b^	0.41^c^
Positive expectations regarding aging	7.3 (1.8)	0.26^b^	0.03^f^
Social connectedness	0 (1.0)	0.49^b^	0.10^c^
Religiosity or spirituality	0 (1.0)	0.20^b^	0.01
Altruism	0 (1.0)	0.28^a^	−0.02

Abbreviation: NA, not applicable.

^a^ Overall model: *F*_13,2,243_ = 184.57; *P* < .001.

^b^ Significant associations after Bonferroni correction (*P* < .002).

^c^ *P* < .001.

^d^ The “other, non-Hispanic” race and ethnicity group included American Indian or Alaska Native, Asian, Native Hawaiian or Other Pacific Islander, and multiracial veterans.

^e^ *P* < .01.

^f^ *P* < .05.

**Figure.**
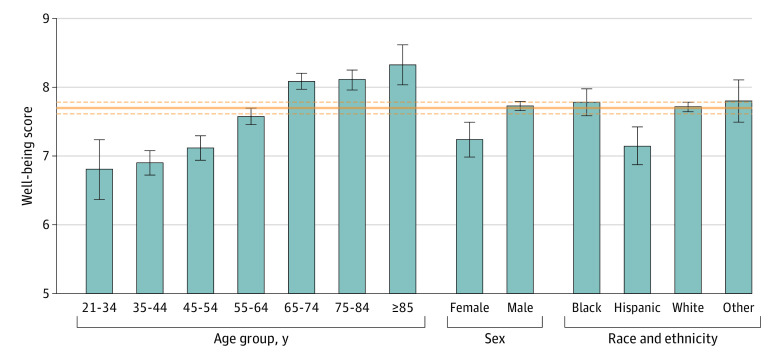
Well-Being Scores Among US Veterans by Age Group, Sex, and Race and Ethnicity Age group (*F* = 37.57; *P* < .001), sex (*F* = 13.37; *P* < .001), and race and ethnicity (*F* = 5.45; *P* < .001) were significantly associated with well-being scores. The orange solid line indicates the mean well-being score in the full sample, and the dashed orange lines indicate the 95% CI. Effect size differences (Cohen *d*) between 65 years or older and 55 to 64 years relative to 21 to 54 years: *d* = 0.77 and *d* = 0.36, respectively; and between 65 years or older and 55 to 64 years: *d* = 0.35. Effect size difference between male and female veterans: *d* = 0.31. Effect size difference between Hispanic and White, Black, and other race and ethnicity groups: *d* = 0.27, 0.28, and 0.27, respectively. Error bars also indicate 95% CI.

The strongest correlates of well-being were protective psychosocial characteristics (β = 0.41), physical health conditions (β = −0.14), and greater age (β = 0.19). Purpose in life moderated the negative association of the number of physical health conditions with well-being (β = 0.25; *P* < .001). Among veterans with a greater number of physical health conditions, those with a higher purpose in life scored substantially higher (effect size difference, Cohen *d* > 1.80) than those with lower purpose in life.

## Discussion

To our knowledge, this study is the first to examine ratings and correlates of well-being in a nationally representative sample of US veterans. The positive age gradient in well-being ratings aligns with a recent study using the same scale in the general US adult population.^3^ This finding could also be explained in part by greater life stressors (eg, burnout, financial instability)^4^ and prevalence of trauma and psychiatric disorders among younger veterans.^5^ Furthermore, lower well-being ratings among female and Hispanic veterans may be partly explained by these subpopulations being overrepresented among younger veterans.^5^

A composite measure of protective psychosocial factors (ie, purpose in life, grit, resilience) was the strongest correlate of well-being, and purpose in life moderated the negative association of physical health conditions with well-being. Furthermore, having fewer physical health difficulties and greater emotional stability and social connectedness were strongly correlated with overall well-being, which suggests that interventions targeting these clinical and psychosocial factors may have potential utility in enhancing well-being in veterans.

Limitations of this study include the cross-sectional design and the use of self-reported measures. Further research is needed to examine whether correlates of well-being may differ between veterans and nonveterans and whether Whole Health interventions designed to increase meaning and purpose in life^6^ may help bolster well-being among veterans.

## Notes

Supplement 1.
eMethods.
**eTable.** Study Measures
eReferences.

Supplement 2.
Data Sharing Statement

## References

[1] National Academies of Sciences Engineering, and Medician. Achieving Whole Health: A New Approach for Veterans and the Nation. National Academies Press; 2023. 37184190

[2] Implementing a Whole Health system: patient and team perspectives. U.S. Department of Veterans Affairs. Last updated March 23, 2022. Accessed June 16, 2023. https://www.va.gov/WHOLEHEALTHLIBRARY/overviews/implementing-a-whole-health-system.asp

[3] Chen Y, Cowden RG, Fulks J, Plake JF, VanderWeele TJ. National data on age gradients in well-being among US adults. JAMA Psychiatry. 2022;79(10):1046-1047. doi:10.1001/jamapsychiatry.2022.2473 36001311 PMC9403847

[4] Bryan CJ, Bryan AO, Baker JC, Ammendola E, Szeto E. Burnout, surface acting, and suicidal ideation among military personnel: results of a longitudinal cohort study. J Soc Clin Psychol. 2022;41(6):593-610. doi:10.1521/jscp.2022.41.6.593

[5] Na PJ, Schnurr PP, Pietrzak RH. Mental health of U.S. combat veterans by war era: results from the National Health and Resilience in Veterans Study. J Psychiatr Res. 2023;158:36-40. doi:10.1016/j.jpsychires.2022.12.019 36565542 PMC11929138

[6] Manco N, Hamby S. A meta-analytic review of interventions that promote meaning in life. Am J Health Promot. 2021;35(6):866-873. doi:10.1177/0890117121995736 33626890

