# Supplementary material for: Well-Being of US Military Veterans
Source: JAMA Netw Open. 2023 Dec 7;6(12):e2346709. doi: 10.1001/jamanetworkopen.2023.46709 (PMC10704281; doi:10.1001/jamanetworkopen.2023.46709)
Supplement: Supplement 1. — eMethods. eTable. Study Measures eReferences. [file jamanetwopen-e2346709-s001.pdf]

# Supplemental Online Content

Na PJ, Fischer IC, Krist AH, Kudler HS, Jeste DV, Pietrzak RH.. Well-being of US military veterans. *JAMA Netw Open*. 2023;6(12):e2346709. doi:10.1001/jamanetworkopen.2023.46709

**eMethods.**
**eTable.** Study Measures
**eReferences.**

This supplemental material has been provided by the authors to give readers additional information about their work.

© 2023 Na PJ et al. *JAMA Network Open.*

**eMethods**

*Participants*

The NHRVS sample was drawn from KnowledgePanel, a research panel of more than 50,000 households that is maintained by Ipsos, a survey research firm. KnowledgePanel® is a probability-based, online non-volunteer access survey panel of a nationally representative sample of U.S. adults that covers approximately 98% of U.S. households. Panel members are recruited through national random samples, originally by telephone and now almost entirely by postal mail. Households are provided with access to the Internet and computer hardware if needed. KnowledgePanel® recruitment uses dual sampling frames that include both listed and unlisted telephone numbers, telephone and non-telephone households, and cell-phone-only households, as well as households with and without Internet access.

Demographic data of survey panel members are assessed regularly by Ipsos using the same set of questions used by the U.S. Census Bureau. Race/ethnicity was assessed via self-report using a standard set of questions used by the U.S. Census Bureau (Black, non-Hispanic; Hispanic; White, non-Hispanic; 2+ Races, non-Hispanic; Other, non-Hispanic). 2+ Races and Other, non-Hispanic were combined. This information was assessed in the current study to characterize the demographic composition of the sample and to adjust for any influence of race/ethnicity in multivariable models.

To permit generalizability of study results to the entire population of U.S. veterans, the Ipsos statistical team computed post-stratification weights using the following benchmark distributions of U.S. military veterans from the most recent (August 2021) Current Veteran Population Supplemental Survey of the U.S. Census Bureau's American Community Survey: age, gender, race/ethnicity, Census Region, metropolitan status, education, household income, branch of service, and years in service. An iterative proportional fitting (raking) procedure was used to produce the final post-stratification weights.

All participants provided informed consent and the study was approved by the Human Subjects Committee of the VA Connecticut Healthcare System.

© 2023 Na PJ et al. *JAMA Network Open.*

## eTable. Study Measures

| Variable | Assessment |
|---|---|
| **Military characteristics** | Enlistment status (enlisted/commissioned, drafted), Combat veteran status (previously deployed, not), years in military, rank/pay grade in military |
| Positive effect of military on life | Rating on the item: “How has being in the military affected your life?” Score range: 1-7 (1=strong negative effect; 7=strong positive effect) |
| Physical health difficulties | Factor score of sum of number of medical conditions endorsed in response to question: “Has a doctor or healthcare professional ever told you that you have any of the following medical conditions?” (e.g., arthritis, cancer, diabetes, heart disease, asthma, kidney disease). Range: 0-24 conditions; disability in activities of daily living[1]: “At the present time, do you need help from another person to do the following?” (e.g., bathe; walk around your home or apartment; get in and out of chair); disability in instrumental activities of daily living: “At the present time, do you need help from another person to do the following?” (e.g., pay bills or manage money; prepare bills; get dressed); and Score on Somatization subscale of the Brief Symptom Inventory-18.[2] |
| Physical exercise | Score on Godin Leisure-Time Exercise Questionnaire.[3] |
| Adverse childhood experiences | Adverse Childhood Experiences Questionnaire[4] total score. |
| Cumulative trauma burden | Life Events Checklist for DSM-5 total score.[5] |
| Military sexual trauma (MST) | Endorsement of either of two items from the VHA MST screen assessing for exposure to military sexual harassment (MSH) and military sexual assault (MSA) was considered a positive screen for MST. MSH was assessed using an item which asked, “When you were in the military, did you ever receive unwanted, threatening, or repeated sexual attention?” MSA was assessed using an item which asked, “When you were in the military, did you have sexual contact against your will or when you were unable to say no?” |
| Lifetime posttraumatic stress disorder (PTSD) | Score of ≥33 on PTSD Checklist for DSM-5,[14] modified to assess lifetime ratings of PTSD symptoms in relation to “worst” Criterion A trauma on the Life-Events Checklist-5 (LEC-5).[15] |
| Lifetime major depressive disorder (MDD) | Positive screen for MDD on the MDD module of the Mini International Neuropsychiatric Interview.[9] |
| Lifetime suicide attempt | Response to question, “Have you ever tried to kill yourself?” |
| Lifetime alcohol use disorder (AUD) | Modified self-report version of AUD module from the DSM-5 version of the Mini Neuropsychiatric Interview.[19] |
| Lifetime drug use disorder (DUD) | Modified self-report version of DUD module from the DSM-5 version of the Mini Neuropsychiatric Interview.[19] |
| Lifetime nicotine use disorder | Score ≥ 5 on the Fagerström Test for Nicotine Dependence (FTND) scale was considered a positive screen.[20] |
| ***Personality traits*** | |
| Extraversion | Score on the extraversion subscale of the Ten-Item Personality Inventory.[5] |
| Agreeableness | Score on the agreeableness subscale of the Ten-Item Personality Inventory.[5] |
| Conscientiousness | Score on the conscientiousness subscale of the Ten-Item Personality Inventory.[5] |
| Emotional stability | Score on the emotional stability subscale of the Ten-Item Personality Inventory.[5] |
| Openness to experiences | Score on the openness to experience subscale of the Ten-Item Personality Inventory.[5] |
| ***Psychosocial factors*** | |

© 2023 Na PJ et al. *JAMA Network Open.*

| | |
|---|---|
| Protective psychosocial characteristics | A composite score of adaptive psychosocial traits[6,7] was used to assess dispositional attitudes and capacities for coping that are associated with more positive mental health outcomes, including qualities such as resilience; a sense of life purpose; dispositional gratitude, optimism, and curiosity/exploration; and perceived community integration. Resilience was measured using the Connor-Davidson Resilience Scale,[8] a 10-item scale with items such as "I am able to adapt when changes occur," measured on a scale from 1 ("not at all") to 5 ("nearly true all the time"); Cronbach's α=0.93. The Purpose in Life Test, Short Form,[9] a 4-item scale, was used to index sense of meaning and purposefulness in life, assessed on a scale from 1 ("no goals/purpose/progress/meaning") to 7 ("very clear goals/purpose/progress/meaning"; Cronbach's α=0.89). Dispositional gratitude, optimism, and curiosity were each assessed using single 7-point Likert scale items adapted from the Gratitude Questionnaire (GQ-6)[10]; the Life Orientation Test-Revised (LOTS-R)[11]; and the Curiosity and Exploration Inventory-II (CEI-II)[12], respectively. Sense of community integration and acceptance was assessed with a single item, "I feel well integrated in my community." |
| Positive expectations regarding aging | Sum score of the following 3 items from the Expectations Regarding Aging scale[13]: "Every year that people age, their energy levels go down a little more;" "It is normal to be depressed when you are old;" and "Forgetfulness is a natural occurrence just from growing old." |
| Social connectedness | Score on 5-item version of the Medical Outcomes Study Social Support Scale[14,15] |
| Religiosity/spirituality | Score on the Duke University Religion Index.[19] |
| Altruism | Factor score of response to the question: "How many days per week do you typically engage in volunteering?" and Frequency of engagement in helping others with instrumental activities of daily living: "How often have you helped a friend, neighbor, or relative other than your spouse or partner with errands, child care, housework, transportation, or other tasks in the past year?" (Response options: Never, 1 to 10 times, 11-50 times, 51-99 times, and 100 or more times[16]). |

Note. DSM=the Diagnostic and Statistical Manual of Mental Disorders

© 2023 Na PJ et al. *JAMA Network Open.*

**References.**


1. Hardy SE, Gill TM. Recovery from disability among community-dwelling older persons. *JAMA.* 2004;291:1596-1602.
2. Derogatis LR. *Brief Symptom Inventroy 18 (BSI-18): Administration, scoring and procedures manual.* Minneapolis, MN: NCS Pearson, Inc.; 2001.
3. Godin G. The Godin-Shephard leisure-time physical activity questionnaire. *Health Fit J Canada.* 2011;4(1):18-22.
4. Felitti VJ, Anda RF, Nordenberg D, et al. Relationship of childhood abuse and household dysfunction to many of the leading causes of death in adults. The Adverse Childhood Experiences (ACE) Study. *Am J Prev Med.* 1998;14(4):245-258.
5. Weathers F, Blake DD, Schnurr PP, Kaloupek DG, Marx BP, Keane TM. The Life Events Checklist for DSM-5 (LEC-5). Instrument available from the National Center for PTSD at www.ptsd.va.gov. 2013. Accessed July 13th, 2022.
6. Pietrzak RH, Cook JM. Psychological resilience in older US veterans: results from the national health and resilience in veterans study. *Depression and anxiety.* 2013;30(5):432-443.
7. Smith NB, Mota N, Tsai J, et al. Nature and determinants of suicidal ideation among US veterans: Results from the national health and resilience in veterans study. *Journal of Affective Disorders.* 2016;197:66-73.
8. Campbell-Sills L, Stein MB. Psychometric analysis and refinement of the connor–davidson resilience scale (CD-RISC): Validation of a 10-item measure of resilience. *Journal of Traumatic Stress: Official Publication of The International Society for Traumatic Stress Studies.* 2007;20(6):1019-1028.
9. Schulenberg SE, Schnetzer LW, Buchanan EM. The purpose in life test-short form: development and psychometric support. *Journal of Happiness Studies.* 2011;12(5):861-876.
10. McCullough ME, Emmons RA, Tsang J. The grateful disposition: a conceptual and empirical topography. *J Pers Soc Psychol.* 2002;82:112-127.
11. Glaesmer H, Rief W, Martin A, et al. Psychometric properties and population-based norms of the Life Orientation Test Revised (LOT-R). *British journal of health psychology.* 2012;17(2):432-445.
12. Kashdan TB, Gallagher MW, Silvia PJ, et al. The Curiosity and Exploration Inventory-II: Development, factor structure, and psychometrics. *J Res Pers.* 2009;43(6):987-998.
13. Sarkisian CA, Steers WN, Hays RD, Mangione CM. Development of the 12-item Expectations Regarding Aging Survey. *Gerontologist.* 2005;45(2):240-248.
14. Sherbourne CD, Stewart AL. The MOS social support survey. *Soc Sci Med.* 1991;32:705-714.
15. Amstadter AB, Begle AM, Cisler JM, Hernandez MA, Muzzy W, Acierno R. Prevalence and correlates of poor self-rated health in the United States: The national elder mistreatment study. *Am J Geriatr Psychiatry.* 2011;18(7):615-623.
16. Brown SL, Nesse RM, Vinokur AD, Smith DM. Providing social support may be more beneficial than receiving it: Results from a prospective study of mortality. *Psychol Sci.* 2003;14(4):320-327.


© 2023 Na PJ et al. *JAMA Network Open.*
