# Supplementary material for: Well-Being of US Military Veterans
Source: JAMA Netw Open. 2023 Dec 7;6(12):e2346709. doi: 10.1001/jamanetworkopen.2023.46709 (PMC10704281; doi:10.1001/jamanetworkopen.2023.46709)
Supplement: Supplement 2. — Data Sharing Statement [file jamanetwopen-e2346709-s002.pdf]

Data Sharing Statement

Na. Well-Being of US Military Veterans. *JAMA Netw Open*. Published December 07, 2023.
doi:10.1001/jamanetworkopen.2023.46709

Data
Data available: Yes
Data types: Deidentified participant data
How to access data: Data will be provided upon reasonable request to rhpietrzak@gmail.com.
When available: With publication

Supporting Documents
Document types: Informed consent form
How to access documents: Data will be provided upon reasonable request to rhpietrzak@gmail.com.
When available: With publication

Additional Information
Who can access the data: Data will be provided upon reasonable request to rhpietrzak@gmail.com.
Types of analyses: Data will be provided upon reasonable request to rhpietrzak@gmail.com.
Mechanisms of data availability: Data will be provided upon reasonable request to rhpietrzak@gmail.com.
